# Adiposity affects emotional information processing

**DOI:** 10.3389/fpsyg.2022.879065

**Published:** 2022-09-26

**Authors:** César Romero-Rebollar, Leonor García-Gómez, Mario G. Báez-Yáñez, Ruth Gutiérrez-Aguilar, Gustavo Pacheco-López

**Affiliations:** ^1^Health Sciences Department, Metropolitan Autonomous University (UAM), Lerma, Mexico; ^2^School of Psychology, Intercontinental University (UIC), Mexico City, Mexico; ^3^Department of Research on Smoking and COPD, National Institute of Respiratory Diseases (INER) Ismael Cosío Villegas, Mexico City, Mexico; ^4^Radiology Department, University Medical Center Utrecht, Utrecht, Netherlands; ^5^Division of Research, School of Medicine, National Autonomous University of Mexico (UNAM), Mexico City, Mexico; ^6^Laboratory of Metabolic Diseases: Obesity and Diabetes, Hospital Infantil de México “Federico Gómez”, Mexico City, Mexico

**Keywords:** adiposity, overweight, obesity, emotional information processing, facial emotion recognition, emotional experience, attentional unawareness, sensory unawareness

## Abstract

Obesity is a worldwide epidemic associated with severe health and psychological wellbeing impairments expressed by an increased prevalence of affective disorders. Emotional dysfunction is important due to its effect on social performance. The aim of the present narrative review is to provide a general overview of human research exploring emotional information processing in overweight and obese people. Evidence suggests that obesity is associated with an attenuation of emotional experience, contradictory findings about emotion recognition, and scarce research about automatic emotional information processing. Finally, we made some concluding considerations for future research on emotional information processing in overweight and obese people.

## Introduction

The increase in the worldwide prevalence of overweight (Body Mass Index: BMI > 24.9) and obesity (BMI > 30) is a major topic of interest due to its association with deleterious health outcomes, including cardiac ischemia, type-2 diabetes, insulin resistance, dyslipidemia, and certain forms of cancer, among other chronic diseases (World Health Organization, 2010; Schulze et al., 2016). The World Health Organization (WHO) estimated that in 2016 globally more than 1.9 billion adults (approximately 39%) were overweight and more than 650 million (approximately 13%) were obese; additionally, it was estimated that more than 340 million of children and adolescents were overweight and obese (World Health Organization, 2021). In this context, there are estimates that up to 35% of the population of countries like the United States, Mexico, and the United Kingdom will be obese by 2030 (OECD/EU, 2017).

Overweight and obesity have been frequently associated with mood disorders, such as anxiety (Amiri and Behnezhad, 2019) and depression (Luppino et al., 2010; Pereira-Miranda et al., 2017). On the contrary, response disruption to emotional stimuli, in the context of cognitive evaluation, is a core feature of mood disorders (Elliott et al., 2011). Thus, being overweight and obese could be associated with emotional information processing impairments.

Emotional information processing is a theoretical framework to assess the organization of cognitive processing in contexts requiring the cognitive evaluation of emotional stimuli (Marsella et al., 2010; Elliott et al., 2011). In this regard, two levels of emotional information processing, namely, explicit and implicit levels, have been suggested (Lane, 2008; Marsella et al., 2010; Moors, 2016; but also see Williams et al., 2008, for a comprehensive review of the *INTEGRATE* model). On the one hand, the explicit level of emotional information processing represents the understanding of the emotional stimuli; it requires a higher level of awareness about the presence of the processed stimuli. This explicit level is assessed through tasks in which the subject has to actively attend, categorize, and recognize the emotional information. For example, an emotional face is presented, and the subject has to correctly identify the displayed emotion (Lane, 2008; Williams et al., 2008; Elliott et al., 2011). In addition, the reported feelings by the subject or subjective emotional experience are considered a component of explicit emotional information processing (Williams et al., 2008). Self-report questionnaires are frequently used to measure subjective emotional experiences, such as the Levels of Emotional Awareness Scale (LEAS; Lane et al., 1990), which assesses the ability to describe one’s own feelings and those of others, and emotional experience impairments through questionnaires such as the Toronto Alexithymia Scale (Bagby et al., 1994b; TAS: Bagby et al., 1994a), which assesses alexithymia, or the difficulties in describing and identifying feelings. Emotion perception tasks are also used to measure subjective emotional experience; in these tasks, affective pictures derived from standardized datasets, such as the International Affective Picture System (IAPS; Lang et al., 2008), are presented to induce emotional states, and the subject has to rate his or her emotional experience in terms of valence, arousal, and dominance. Other forms of emotional induction are through video clips and music (Elliott et al., 2011). However, to the best of our knowledge, there are no published papers about the induction of emotional experiences using video clips or music in obese subjects, thus only studies using IAPS were included in this review.

On the other hand, implicit or automatic processing of emotions represents the ability to evaluate emotional stimuli without awareness of the emotional signal (Elliott et al., 2011; Diano et al., 2017). The common way to assess implicit affective processing is through sensory unawareness tasks and attentional unawareness tasks. For example, in sensory unawareness tasks, the emotional stimulus fails to enter awareness due to the suppression of the normal perceptual processing, and in affective priming tasks, the subliminal exposure to a valenced stimulus, called prime, unintentionally influences the evaluation and classification of a subsequent valenced stimulus, called target (Klauer and Musch, 2003). In attentional unawareness tasks, the emotional stimulus is accessible but the subject’s attention is focused on another aspect different from emotional content, for example, the subject has to name the color of an affective word instead of reading it, or judge the gender of an emotional face instead of the emotion (Elliott et al., 2011; Diano et al., 2017).

The aim of the present narrative review is to provide a short but up-to-date general overview of human studies in which emotional information processing features in overweight and obese samples were explored. First, we summarized self-reported and neurocognitive evidence related to the explicit level of emotional information processing. Then, we reviewed neurocognitive literature about implicit emotional information processing. Finally, we made some concluding considerations for future research on emotional processing in overweight and obesity.

### Explicit emotional information processing

#### Overweight/obesity and emotion recognition

**FIGURE 1:**
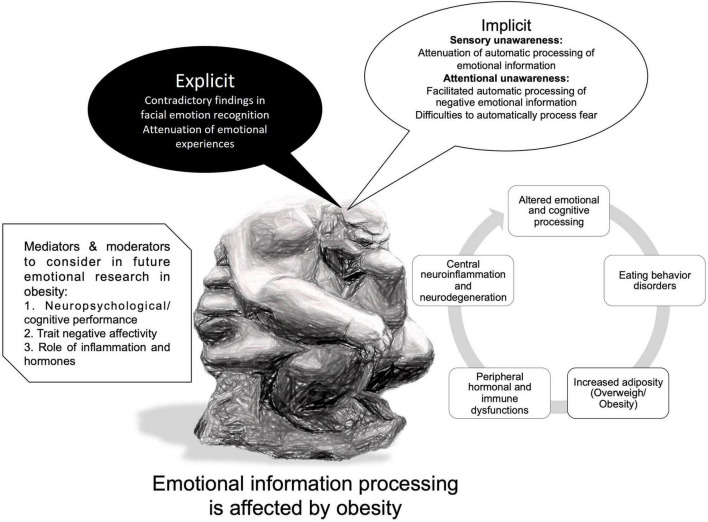
Summary of findings of emotional information processing in overweight and obesity.

The “Explicit emotional information processing” and “Implicit emotional information processing” sections of this review contain the findings about emotional information processing in overweight/obesity, methodological details, and the main results of reviewed studies are summarized in Table 1 and depicted graphically in Figure 1.

**TABLE 1:** Main characteristics of reviewed studies.

References	Sample	Emotional information processing measures	Outcome	Results
*Explicit emotional information processing: emotion recognition*				
Baldaro et al. (1996)	Children *Males and Females* (Age: 8–16 years) Groups: Developmental obesity group (BMI: 10/30% more than the correct weight, *n* = 20) Normal weight group (BMI: correct weight, *n* = 20)	42 photos of seven facial expressions: Anger Sadness, Disgust, Surprise, Fear, Happiness and Neutral	Number of emotion recognition errors: total and by emotion facial expressions	Obesity group: Higher total recognition errors Higher recognition errors of happiness and neutral facial expressions
Koch and Pollatos (2015)	Children *Males and Females* (Age: 6–10 years) Groups: Overweight/Obesity group (mean BMI = 22.61, > 90th BMI percentile, based on the national reference data for German children; *n* = 33) Normal weight group (mean BMI = 16.23, ≥ 25th to ≤ 75th BMI percentile, based on the national reference data for German children; *n* = 33)	20 photos of four facial expressions: Happiness, Anger, Sadness, and Neutral	Hit rate (%): total and by emotion facial expressions Reaction time: total and by emotion facial expressions	Overweight/Obesity group: Lower total hit rate Increased total reaction time
Percinel et al. (2018)	Children and adolescents *Males and Females* (Age: 11–18) Groups: Obesity group (mean BMI = 34, > 95th BMI percentile, based on reference for Turkish children; *n* = 30) Normal weight group (mean BMI = 20.3, ≥ 25th to ≤ 75th BMI percentile based on reference for Turkish children; *n* = 30)	Faces test: 60 photos of six facial expressions: Happiness, Sadness, Surprise, Anger, Disgust, and Fear	Total correct recognition answers	Obesity group: Lower total correct recognition answers
Turan et al. (2019)	Adolescents *Males and Females* (Age range not reported) Groups: Obesity + BED group: (mean BMI = 34.58, >95th BMI percentile, reference was not mentioned; *n* = 32) Obesity non-BED group: (mean BMI = 32.72, > 95th BMI percentile, reference was not mentioned; *n* = 32) Normal weight group: (mean BMI = 20, ≥ 3rd to ≤ 85th BMI percentile, reference was not mentioned; *n* = 64)	Faces test: 18 photos of six facial expressions: Happiness, Sadness, Surprise, Anger, Disgust, and Fear	Total correct recognition answers	Obesity groups: Lower total correct recognition answers irrespective of their BED status
Bergmann et al. (2016)	Adults *Females* (Age range not reported) Groups: Obesity group (BMI ≥ 30; *n* = 73). Normal weight group (BMI ≤ 24.9; *n* = 73)	42 photos of eight facial expressions: Anger, Sadness, Disgust, Surprise, Fear, Happiness, Contempt, and Neutral	Total correct recognition answers	No differences between obesity and normal weight groups
Surcinelli et al. (2007)	Preadolescents and adolescents *Males and Females* (mean age: 12.3 years) Groups: Obesity group (BMI: boys scored between 1.63 and 2.45 standard deviations and girls between 1.74 and 2.64 standard deviations from the 50th percentile of the population of Italian children; *n* = 30) Normal weight group (BMI: boys scored between -1.10 and 0.54 standard deviations and girls scored between -1.27 and 1.08 standard deviations from the 50th percentile of the population of Italian children; *n* = 30)	42 photos of seven facial expressions: Anger, Sadness, Disgust, Surprise, Fear, Happiness, and Neutral	Total correct recognition answers	No differences between obesity and normal weight groups
*Explicit emotional information processing: emotional experience*				
Rommel et al. (2012)	Adults *Women* (Age range not reported). Obesity group (mean BMI = 39.16; *n* = 94). Normal weight group (mean BMI = 22.61; *n* = 56)	Levels of Emotional Awareness Scale (LEAS)	LEAS total score LEAS Self LEAS others	Obesity group: Attenuation of emotional experience expressed as lower scores in three subscales of LEAS
Surcinelli et al. (2007)	Described above	Levels of Emotional Awareness Scale (LEAS)	LEAS total score	Obesity group: Attenuation of emotional experience expressed as lower LEAS total score
Giel et al. (2016)	Adults (Age range no reported) Study 1 *Women* Groups: Obesity group (mean BMI = 43.2; *n* = 33) Normal weight group (mean BMI = 21.3; *n* = 25) Study 2 *Men* Groups: Obesity group (mean BMI = 37.4; *n* = 29). Normal weight group (mean BMI = 24.1; *n* = 29)	Emotional perception task: Pictures from the International Affective Picture System (IAPS) of six emotional conditions (fear, anger, sadness, happiness, fear-anger, fear-sadness). The subject have to rate each picture in three dimensions: valence, arousal and dominance	Ratings of valence, arousal and dominance of each emotional condition	Obesity group: Attenuation in emotional experience expressed as lower values in arousal and dominance in emotional conditions
*Implicit emotional information processing: Sensory unawareness*				
Cserjési et al. (2011)	Adults *Females* (Age range not reported) Groups: Obesity group (mean BMI = 34.2; *n* = 30) Normal weight group (mean BMI = 22.8; *n* = 30)	Affective priming task: Stimuli: Prime: schematic emotional faces (happy, neutral, sad, angry) Target: positive and negative adjectives Conditions: Affectively congruent (prime/target): +/+ or -/- Affectively incongruent (prime/target): ± or -/+	Affective priming effect (significant difference between facilitation and inhibition effect)	Obesity group: Attenuation in the processing of sad and angry faces expressed as no priming effect
Wegener et al. (2008)	Adults *Men and Women* (Age range not reported) Groups: Obesity group (mean BMI = 38.5; *n* = 30) Normal weight group (mean BMI = 23.4; *n* = 25)	Affective priming task: Stimuli: Prime: Words with positive and negative connotations Target: Words with positive and negative connotations Conditions: Affectively congruent (prime/target): +/+ or -/- Affectively incongruent (prime/target): ± or -/+	Affective priming effect (difference in error rates of target classification between congruent and incongruent conditions)	Obesity group: Attenuation in the automatic affective processing expressed as a smaller affective priming effect
*Implicit emotional information processing: Attentional unawareness*				
Hanson et al. (2020)	Adult U.S. Soldiers *Men and Women* (Age: 19–50) Groups: Obesity group (BMI = 30; *n* = 29) Normal weight group (BMI = 29.99; 80)	Emotional Stroop: series of words of various colors appeared on a screen, the subject must to tapping the color of the word instead of reading the word Stimuli: combat-related words (negative valence)	Number of correct answers	Obesity group: Difficulties to inhibit automatic processing of negative affective information expressed as lower number of correct answers
Scarpina et al. (2021)	Adults *Women* (Age range not reported). Groups: Obesity group (mean BMI = 43.79, BMI > 30; *n* = 20) Normal weight group (mean BMI = 22.17, BMI > 18.5 < 24.9; *n* = 20)	Redundant target task: individuals generally respond faster when two identical targets are presented simultaneously rather than when presented alone; moreover, the competitive presence of a distractor (that is another emotion or a neutral expression) affects the correct recognition of the target Stimuli: photos of facial expressions of anger, fear and neutral, presented in four conditions: (1) Single: the target (i.e., the face expressing the target emotion) was presented on the right OR left of a fixation cross (2) Congruent: the target was presented simultaneously on the right AND left of the fixation cross (3) Emotional incongruent: the target was presented on the right OR left of the fixation cross along with a different emotion (4) Neutral incongruent: the target was presented on the right OR left of the fixation cross along with a neutral expression	Accuracy Reaction time	Obesity group: Facilitated automatic processing of anger Difficulties in automatic processing of fearful faces

BMI, body mass index; BED, binge eating disorder.

The most basic form of explicit emotional information processing is emotion recognition. Emotion recognition tasks (ERT) consist of the presentation of emotional faces displaying basic emotions such as anger, disgust, surprise, fear, happiness, sadness, and neutral expressions. Then, the subject must select the verbal label corresponding to the emotion. The most common measures of performance are global correct responses and errors, correct responses and errors by emotional condition, and reaction time (RT) for correct responses (Elliott et al., 2011).

The results of ERT in overweight and obese people are contradictory. Some studies reported emotion recognition impairments in obesity. Baldaro et al. (1996) studied a group of obese male and female children compared to a normal weight group. They reported that the obesity group had higher global emotion recognition errors for the entire task and happiness and neutral conditions (refer to Table 1). Koch and Pollatos (2015) reported lower global emotion recognition accuracy and increased global RT for correct responses, which represent difficulties in recognizing emotions in obese children compared to a normal weight control group (refer to Table 1). Similar difficulties in recognizing emotions in obesity were reported by Percinel et al. (2018) in another study carried out in male and female obese adolescents compared to a normal weight group (refer to Table 1). Turan et al. (2019) compared the global accuracy in an emotion recognition task between three groups of adolescents: (1) obesity plus binge eating disorder (BED) group; (2) obesity without BED group; and (3) normal weight group. The authors reported lower global emotion recognition accuracy in both obesity groups compared to the normal weight group (refer to Table 1).

In contrast, a couple of studies reported no association between emotion recognition impairments and obesity. Surcinelli et al. (2007) reported no difficulties in recognizing emotions in obese preadolescents and adolescents compared to a normal weight control group (refer to Table 1). Likewise, Bergmann et al. (2016) reported similar results of no differences in global emotion recognition accuracy between obese adult women and the normal weight control group (refer to Table 1).

#### Overweight/obesity, subjective emotional experience, and emotion perception

Subjective emotional experience is a component of explicit emotional information processing, and it is mainly measured through self-reported questionnaires, such as LEAS and TAS, and through affective ratings of emotional perception.

The finding about the attenuation of subjective emotional experience in obesity is consistent. Regarding self-reported questionnaires, attenuation of subjective emotional experience can be inferred if a person gets a low score in LEAS or a high score in TAS. Surcinelli et al. (2007) reported lower total LEAS scores in obese pubescent and adolescents, compared to a normal weight group (refer to Table 1 for details). Similarly, Rommel et al. (2012) found lower scores in the three subscales of LEAS in obese adult women (refer to Table 1 for details). Consistently, a meta-analysis of 13 studies of alexithymia in obese subjects reported increases in the three subscales of TAS in obese samples and provides robust evidence about a general attenuation of subjective emotional experience (Fernandes et al., 2018).

As previously mentioned, emotion perception is a component of emotional experience. In emotion perception tasks, an affective stimulus is presented, for instance, a pleasant picture. Then, the subject rates its own emotional experience elicited by the previously seen stimuli in three dimensions: (i) *valence;* evaluation of how pleasant is the emotional stimulus for me; (ii) *arousal or intensity;* how excited I feel by the emotional stimulus; and (iii) *dominance or potency/control;* how controlled I feel by the emotional stimulus (Sutton et al., 2019). In this context, Giel et al. (2016) reported that obese men and women had lower scores than normal-weight people in arousal and dominance dimensions in an emotion perception task (refer to Table 1).

### Implicit emotional information processing

Diano et al. (2017) proposed a useful theoretical framework to distinguish between two implicit emotional information processes: sensory unawareness and attentional unawareness.

#### Overweight/obesity and sensory unawareness

*Sensory unawareness* refers to being unaware of a stimulus due to its “subliminal presentation,” that is, the stimulus is presented rapidly or the intensity of the stimulus is below the detection threshold, provoking a failure to consciously report the presence of the stimulus (Diano et al., 2017). Affective priming tasks are commonly used to elicit sensory unawareness and to assess automatic evaluative processes. In brief, affective priming tasks assess whether the evaluation of a first stimulus, called the prime, that contains positive or negative emotional information and is rapidly presented to get ignored by the subject, affects the processing of subsequent stimuli, called the target. Performance indexes based on reaction time to targets represent two effects: (1) the prime facilitates the response to the target, called the *facilitation effect*; and (2) the prime interferes with the response to the target, called the *inhibition effect*. If both effects are statistically different, there is a priming effect that implies that the emotional information, positive or negative, is automatically processed (Diano et al., 2017; see Klauer and Musch, 2003 for a comprehensive review of affective priming tasks).

Regarding sensory unawareness in overweight and obesity, Wegener et al. (2008) reported an attenuation of automatic emotional processing in a group of obese adults. The authors designed an affective priming task using words as the prime and target stimulus (refer to Table 1). Similarly, Cserjési et al. (2011) reported an attenuation of implicit processing of sad and angry human faces in a group of obese adult females. The affective priming task’s stimuli were schematic emotional faces as prime and word-adjectives as target stimuli (refer to Table 1).

#### Overweight/obesity and attentional unawareness

*Attentional unawareness* refers to the cognitive processing of a stimulus that is outside of the attentional focus. It is assumed that emotional stimulus, automatically and unconsciously, attracts the subject’s attention, and thus the subject must suppress the automatic allocation of attention to accomplish the task’s goals (Diano et al., 2017). Some cognitive tasks have been used in emotion research to assess attentional unawareness: (a) “Emotional Stroop” in which a series of colored affective words are presented and the subject is instructed to name the color rather than the word. (b) “Dual-task performance” implies the resolution of a cognitively demanding task while a task-irrelevant emotional stimulus is presented outside of the attentional focus. (c) “Non-emotional judgment tasks” in which subjects make judgments based on other features of emotional stimuli instead of the emotional content; for instance, if an emotional face is presented, the subject is instructed to recognize the gender instead of emotion. (d) “Redundant-target tasks” in which the subject is instructed to detect an emotional target when a neutral distractor is present (Elliott et al., 2011; Diano et al., 2017).

Research about attentional unawareness in overweight and obese people is scarce, and the task design varies across studies. However, facilitated automatic processing of aversive information in general (Hanson et al., 2020) has been observed in obesity; anger in particular (Scarpina et al., 2021). Additionally, Hanson et al. (2020) designed an “emotional Stroop task” (see Williams et al., 1996) using combat-related words as negative emotional stimuli. The authors assessed adult soldiers divided into an obesity group and a normal weight control group. The obesity group presented lower correct answers in the task, which represent difficulties in inhibiting automatic processing of negative affective information (refer to Table 1). Furthermore, Scarpina et al. (2021) designed a redundant target task using fearful, angry, and neutral facial expressions as stimuli. The authors reported a dissociated effect in the automatic processing of negative emotional information. That is, obese women, compared to a normal weight group, presented facilitated automatic processing of anger and difficulties in automatically processing fearful stimuli (refer to Table 1).

## Concluding considerations

We addressed the association between overweight/obesity and characteristics of explicit and implicit emotional information processing. Research about the explicit aspects of emotional information processing (emotion recognition, emotional experience, and emotional perception) is vast compared to the studies that explore implicit aspects (sensory and attentional unawareness). Regarding subjective emotional experiences, self-reported studies are consistent with the attenuation of emotional experiences in obesity. In fact, Fernandes et al. (2018) presented an interesting meta-analysis demonstrating higher values in alexithymia and two components, such as difficulties in identifying feelings and an externally oriented thinking style, which represent impairments or reductions in emotional experience in obesity. In particular, these findings about alexithymia are interesting because it has been suggested that externally oriented thinking disrupts the ability to recognize facial emotions (Lyvers et al., 2018), and higher levels of alexithymia are associated with a suppression strategy to regulate emotions (Swart et al., 2009). Later, we will return to the implication of emotional regulation impairments in the maintenance of overweight/obesity.

Findings about emotion recognition in subjects suffering from overweight/obesity are contradictory. We identified six studies with similar task structures to explore emotion recognition. Four studies reported that obese subjects present difficulties in recognizing emotions based on general recognition accuracy, global errors, and rate of correct responses (Baldaro et al., 1996; Koch and Pollatos, 2015; Percinel et al., 2018; Turan et al., 2019). Of these studies, one reported more errors in the recognition of specific emotions such as happiness and neutral expressions (Baldaro et al., 1996). Thus, more research is needed to explore and confirm the effect of overweight/obesity on the recognition of specific emotions. On the contrary, two emotion recognition studies reported no impairments in overweight and obesity (Surcinelli et al., 2007; Bergmann et al., 2016). The authors argue that the inconsistencies with previous results may be due to the small sample size, inaccurate adiposity classification of subjects, and the presence of psychiatric symptoms that must be measured (Surcinelli et al., 2007). However, Bergmann et al. (2016) suggested that socio-emotional impairment in obesity, such as increased alexithymia, does not rely on explicit face recognition. In addition to those arguments, a study with bariatric surgery obese-candidates (mean BMI = 46.29) reported that neuropsychological performance, such as more planning errors, predicted lower accuracy in emotion recognition, and worst performance in executive attention and memory tasks predicted slower reaction times for corrected recognized emotions (Manderino et al., 2015). Thus, supplementary research is needed to define the possible mediating role of neuropsychological performance in the association between obesity and emotion recognition difficulties.

Regarding scarce research about implicit emotional information processing, the findings about sensory unawareness, assessed through affective priming tasks, suggest an attenuation of automatic affective processing (Wegener et al., 2008) and failure to be attentive to negative emotions (Cserjési et al., 2011). Some considerations should be taken into account: affective priming as a sensorial phenomenon does not rely on attentional mechanisms (see Diano et al., 2017 for an extensive review); thus, the origin and implications of this attenuated automatic affective processing based on sensory features remain unclear and must be fully addressed.

Evidence about attentional unawareness in overweight and obese people suggests privileged access to the cognitive evaluation system of aversive information (Hanson et al., 2020), specifically anger, but not fear (Scarpina et al., 2021). Despite few studies, some insights could be proposed as consistent findings: in obese people, specifically anger but not all negative emotional information is automatically processed. In this context, we consider that to get a better understanding of emotional processing in overweight/obesity, some variables should be taken into account in further research. In particular, individual differences in emotional information processing could be partially explained by the disposition and tendency to experience negative emotions, called “trait negative affectivity” (Cyders and Smith, 2008; Nelis et al., 2016). The rationale for considering trait negative affectivity as a promising variable to evaluate in emotion research in overweight/obese people derives from reports about its increased value in obese samples (Carr et al., 2007; Pasco et al., 2013), and from observations in healthy subjects that negative affect inhibits affective priming effects (Storbeck and Clore, 2008). To the best of our knowledge, there are no studies on overweight and obese subjects exploring the contribution of trait negative affectivity to implicit or explicit emotional information processing. Thus, future emotional processing research in overweight/obesity could include trait negative affectivity measures, which are easily assessed using “The Positive and Negative Affect Schedule” (PANAS: Watson et al., 1988).

Evidence indicates that emotional information processing research in obesity is biased toward negative emotion categories such as anger, fear, and sadness, which may be due to its association with mood disorders and the implications of emotional information processing in socio-affective processes. However, the processing of other emotion categories, specifically disgust, could give us more understanding of the etiology and maintenance of overweight/obesity due to its regulatory function and its protective role in preventing the ingestion of potentially noxious/toxic substances (Hartmann and Siegrist, 2018). In this regard, promising evidence has been reported, as it has been suggested that obesity is associated with less activation in the right insula elicited by contaminated food images (Watkins et al., 2016), lower disgust sensitivity (Houben and Havermans, 2012; Liu et al., 2019), and lower food disgust scores (García-Gómez et al., 2020). Furthermore, an inverse relationship between BMI and moral disgust has been reported (Vicario and Rafal, 2017). The last is particularly interesting because it has been suggested that the evoked facial reaction of disgust is preserved from sensory distaste, disgust processing, and moral transgressions (Chapman et al., 2009). Finally, it is worth noting that inflammation has recently been shown to modulate emotion processing (Schedlowski et al., 2014; Dooley et al., 2018; Lasselin et al., 2020; Hansson et al., 2021), so we hypothesize that chronic low-grade inflammation experienced by overweight subjects, and exacerbated in obesity, might be the mechanism behind the dysfunctions reported in emotion processing.

Considering the evidence that obesity is characterized by attenuation in explicit emotion recognition, attenuation in emotional awareness, increased alexithymia, and attenuation in automatic processing of emotional information related to sensory unawareness, we conclude that overweight/obesity affects emotional information processing. However, there is no evidence that such impairments are an etiological factor of overweight/obesity *per se*; rather, they are a consequence, a cognitive marker of negative mood that triggers weight gain in a vicious cycle. In accordance, Marks (2015) proposed that overweight/obesity is, in part, the result of an imbalance in psychological homeostasis derived from three processes whose central characteristic is negative mood: (1) dissatisfaction with body image generated by negative public perception of weight gain; (2) increase in negative affect that is expressed as anxiety, depression, and stress; and (3) overeating high-fat food and sugary drinks as a behavioral strategy to reduce negative mood. Furthermore, this position is further supported by findings about disruption in the ability to recognize emotions after negative mood induction in healthy populations (Chepenik et al., 2007), and the positive correlation between symptoms of depression and anxiety with difficulties in emotional awareness in non-clinical populations (Sendzik et al., 2017). Notably, alterations in emotional information processing in obesity, mainly alterations in emotional experience, could be considered a maintenance factor of this condition given the effect of these basic emotional skills on emotional regulation processes (Boden and Thompson, 2015). Effective emotional regulation requires the individual’s ability to be aware of their internal emotional states in order to identify the emotion experienced (Raman et al., 2013). In this context, it has been suggested that obese people tend to engage in emotional eating, that is, an increased intake of highly palatable and hypercaloric foods and beverages in order to reduce the discomfort associated with negative emotions and negative mood, which results in the maintenance of high BMI (Raman et al., 2013; Konttinen, 2020). It has been proposed that high levels of emotional awareness may improve emotional recognition in people who have a style of emotional regulation based on suppression (behavioral inhibition of emotional expression) and masking (covering negative emotions with facial expressions of happiness; Szczygieł et al., 2012). In fact, training in identifying and expressing emotions has been used in psychological interventions focused on the management of emotional triggers of overeating in obese subjects with comorbid BED (Torres et al., 2020). This evidence suggests that training in emotional awareness skills could be a plausible therapeutic target for BMI reduction and control.

Finally, it is clear that more research is needed to understand the contribution of variables such as cognitive performance or trait emotionality on emotional information processing and more replication studies, specifically about implicit levels, are needed. Emotional dysfunction is important due to its negative impact on social performance. The exploration of the cognitive processing of emotional information in overweight/obesity could lead us to understand the contribution of cognitive factors in the maintenance of this deleterious condition.

## Author contributions

CR-R, LG-G, and GP-L: conceptualization and writing of the original draft. MB-Y and RG-A: critical review of the final version. All authors contributed to the article and approved the submitted version.
